# Characteristics of Electronic Health Services in Saudi Arabia During the COVID-19 Pandemic

**DOI:** 10.7759/cureus.28441

**Published:** 2022-08-26

**Authors:** Saleh Alqifari, Saleh M Saleh, Osama Habboush, Ahmad A Ibrahim

**Affiliations:** 1 Department of Pharmacy Practice, College of Pharmacy, University of Tabuk, Tabuk, SAU; 2 College of Medicine, Sulaiman Alrajhi University, Qassim, SAU; 3 College of Medicine, Sulaiman AlRajhi University, Qassim, SAU

**Keywords:** covid-19 retro, coronavirus disease 2019, pandemic, e-services, healthcare, covid-19

## Abstract

Introduction: Saudi Arabia’s experience using digital technology during the COVID-19 pandemic helped the country in tackling the pandemic. We aim to explore a bundle of consumer-directed electronic health services released by the Ministry of Health (MOH) in Saudi Arabia in response to the COVID-19 pandemic.

Methods: We reviewed all electronic health services that have been released in response to the COVID-19 pandemic by the MOH in Saudi Arabia. A list of the services has been prepared and each service has been explored in detail. The service terms and procedures have also been reviewed for pertinent information.

Results: There were 13 services noted in total. Services were devoted to the general public and healthcare practitioners. Services ranged from simple appointment booking for the COVID-19 vaccine to interactive maps of all available healthcare centers and electronic prescriptions of medications. In addition, 10 applications were published for use on smartphones.

Conclusion: The digitalization of healthcare services in Saudi Arabia has eased communication between the public and healthcare professionals. Furthermore, electronic health services served as an effective tool against the spread of COVID-19 infection during the pandemic.

## Introduction

The discovery of the novel coronavirus disease (COVID-19) in December 2019 had severe global implications [1]. Daily activity was disrupted as a result of the spread of the disease. To preserve functionality in many parts of life, several risk-reduction strategies were utilized such as applying technology in healthcare to impede the pandemic [2]. Social distancing was the center of all global attempts to contain the pandemic [1,2] While additional preventative measures such as wearing masks, keeping a safe distance, testing exposed or symptomatic individuals, contact tracing, and isolation have helped restrict the spread where these precautions have been strictly followed, immunization was still crucial to reducing COVID-19-related morbidity and mortality [3,4]. Saudi Arabia has taken the initiative in establishing disease containment measures and worked tirelessly to address the needs of the community promptly. The government's mitigation efforts included a bundle of electronic health services which were supported by the availability of technologies such as smartphones, laptops, desktop computers, and tablets [2]. This paper aims to briefly highlight Saudi Arabia’s experience using digital technology during the COVID-19 pandemic.

## Materials and methods

A literature search including official government websites, official media outlets, and official government Twitter accounts was conducted in December 2021 to identify and analyze all electronic health services released by the Saudi Ministry of Health (MOH) in response to the COVID-19 pandemic. A list including all services released has been prepared, and each service has been thoroughly reviewed. Terms and procedures for the services have also been evaluated for pertinent information.

## Results

In response to the COVID-19 pandemic, Saudi Arabia utilized 13 electronic health services and 10 smartphone applications. Some of these services and smartphone applications received were available prior to the COVID-19 pandemic, however, they received additional features as a result of the pandemic. The smartphone applications and services can be categorized by the targeted population, either directed to all community members or healthcare practitioners (Tables 1, 2).

**Table 1:** Ministry of health services for all community members

Service	Description	Mode of delivery
Interactive maps	A free service that facilitates navigation to various health-care centers and provides the working hours for each center by center’s name and region.	MOH website
COVID-19 vaccine approval service in Kingdom	A free service that enables registration of approved COVID-19 vaccines (Pfizer-BioNTech, Moderna, Oxford-AstraZeneca, Sinopharm, Sinovac and Janssen.) taken outside Saudi Arabia.	MOH website
(Efada) Inquiry Service	A free service that allows for receiving COVID-19 test results done in one of MOH’s authorized centers even if not received as SMS.	MOH website
E-Prescription Service	A free service that allows patients to receive medications from nearest pharmacies through consulting MOH's remote channels.	Calling 937-Service Center or using Sehaty smartphone application
(Mawid) Service	A free service that enables users to book, cancel or reschedule their appointment at primary health care centers or referral appointments.	Calling 937-Service Center, using Mawid smartphone application, through MOH website or heading directly to the nearest healthcare center
COVID-19 Vaccine Booking Service	A free service that allows users to book an appointment to receive the COVID-19 vaccine.	Through Sehaty smartphone application
Registration for COVID-19 In-Home Vaccination for 70+ Years.	A free service that allows vaccination at home for people aged more than 70 years.	MOH website
Exemption from COVID-19 Vaccines Service	A free service that allows for temporary or permanent exemption from COVID-19 vaccines for certain people based on medical reports approved by the concerned committee.	MOH website
HESN	Health Electronic Surveillance Network (HESN) is a program that provides public health data including data about the epidemic outbreak, vaccination and immunization.	MOH website
Taqasi	Is a program to trace individuals infected with COVID-19 based on the results obtained from the HESN program.	MOH website

 

**Table 2:** MOH services for healthcare practitioners

Service	Description	Mode of delivery
Hajj Visiting Manpower Participation Service	A free service that enables qualified practitioners who are interested in participating as a manpower supporting hospitals and health care facilities in Makkah and Madinah during Hajj season.	MOH website
(Visitors) Service	A free service that enables healthcare practitioners to temporarily provide their medical services during their visits to Saudi Arabia.	MOH website
Anat	A free service that facilitates communication between healthcare practitioners and sharing important information of the patients.	Anat.sa website or using the Anat smartphone application

Some smartphone applications were in service before the COVID-19 pandemic and were upgraded with additional features during the pandemic (Table 3) [5].

**Table 3:** MOH applications for smartphones

Application name	Description
Sehhaty	A free application that is available on Apple Store or Google Play that allows for 24 hours access to personal health information, booking for appointments to either virtual or in-person counseling, booking for COVID-19 tests and vaccines, searching for the availability of the medication, and finding the nearest pharmacy that carries this medication.
Mawid	A free application available on Apple Store or Google Play that enable users to book, cancel or reschedule their appointment at primary health care centers or referral appointments.
Seha	A free application available on Apple Store or Google Play that enables users to request lab and medical reports and pay for them not only but also allows users to find their family health records.
Seha for Providers​	A free application is available on Apple Store or Google Play that enables doctors to receive medical consultations from other doctors in all specialties.
Tawakkalna	A free application is available on Apple Store or Google Play that was initially designed to show COVID-19 status and is now updated to give various services including permit, health education, COVID-19 vaccination, and health passport which provide personal information, vaccination status, recent COVID-19 test and medical insurance status (Figure 1).
Tabaud	A free application is available on Apple Store or Google Play that allows the users to know if they have contacted a COVID-19 patient in the past 14 days.
Anat	A free application on Apple Store or Google Play that supports communication between providers.
Asefni	A free application is available on the Apple Store or Google Play that facilitates activating the emergency team of Saudi red crescent and provides accurate location.
Tetamman	A free application on the Apple Store or Google Play is directed to contacts of confirmed cases, suspected cases, and confirmed cases to follow up their health status, contact 937-service centers easily, and find health-related educational content.
Labayh	A free application on Apple Store or Google Play that is made to provide easy and safe psychological consultation. The application was very useful during the tough period of the novel COVID-19.

Services were further categorized by their functionality into digital screening, surveillance, contact notification, vaccination, follow-up, and psychological support (Table 4).

**Table 4:** Functions of applications and services

Functionality	Applications & Services Available
Digital screening	Tawakkalna, Mawid, Sehhaty, Interactive maps
Surveillance	Health Electronic Surveillance Network (HESN), Taqasi
Contact notification	Tabaud, Tawakkalna
Vaccination facilitation	Tawakkalna, Sehhaty, Efada Inquiry Service, COVID-19 Vaccine Booking Service, Interactive maps, Registration for COVID-19, In-Home Vaccination for individuals >70-year-old, Exemption from COVID-19 Vaccines Service, COVID-19 vaccine approval service
Follow-up after infection	Tetamman, Tawakkalna, Interactive maps, Electronic Prescription Service, Seha
Psychological support	Labayh

Digital screening help in easing the process of COVID-19 testing and determine the state of the patient as positive, negative, in contact or immune. While surveillance helps to gather data to support future efforts to ease the effect of the pandemic. The contact notification function traces individuals with positive tests and provides notification upon contact with healthy individuals. Groups of applications or services are helping to ease the COVID-19 vaccination process, following up on the health status of people who tested positive or took the vaccine. Some of these applications and services were developed before the pandemic, some were upgraded during the pandemic, and others were developed concomitant to the COVID-19 outbreak [5]. Moreover, these applications played a major role in tackling the pandemic, as they helped tremendously with social distancing by utilizing phone location services to keep users aware of individuals with COVID-19 active infection in the vicinity at all times. Users of these applications and services may log in via the National Single Sign-On service provided by the National Information Center (NIC) which allows users to access all government services using their national or residence identification credentials.

During the pandemic, these applications also made it possible for individuals to make appointments for the COVID-19 test or vaccine on their phones. Individuals then can check all their results directly from their phone, all without the need to go or call any hospital or test center. Official government and private entities can check individuals’ health and vaccine status through official credentials. This provided a trusted system to check on the status of an individual or a group of individuals which later facilitated the return of services such as airline, education and pilgrims. In Saudi Arabia, these applications help in controlling and tackling the spread of COVID-19 infection and decrease the number of confirmed COVID-19 deaths, reaching a staggering 0.03 confirmed deaths per million capita in April 2022. In addition, with the aid of these digital applications, the percentage of the population vaccinated increased significantly reaching 72% fully vaccinated individuals and 77% with at least one vaccine dose in April 2022 [6].

## Discussion

The need for digital transformation in healthcare has been evident. With the COVID-19 pandemic, the need for digitalization prevailed immensely. During the pandemic, digitalization aided in areas such as digital screening, contact tracing, awareness spreading, online appointment booking, a remote medical consultation and follow-up, surveillance, data collection, scientific research, vaccination, and psychological support. During the COVID-19 pandemic, Saudi Arabia has been one of the first adopters of digital public health services as a tool to tackle the spread of the COVID-19 outbreak. Three smartphone applications were released during the COVID-19 pandemic wincludeludes Tetamman, Tawakkalna, and Tabaud. Tetamman was the first application to be published in March 2020 by the MOH for viewing the COVID-19 test results, common signs and symptoms, and a tool to raise public awareness regarding precautions during the pandemic. 

**Figure 1:**
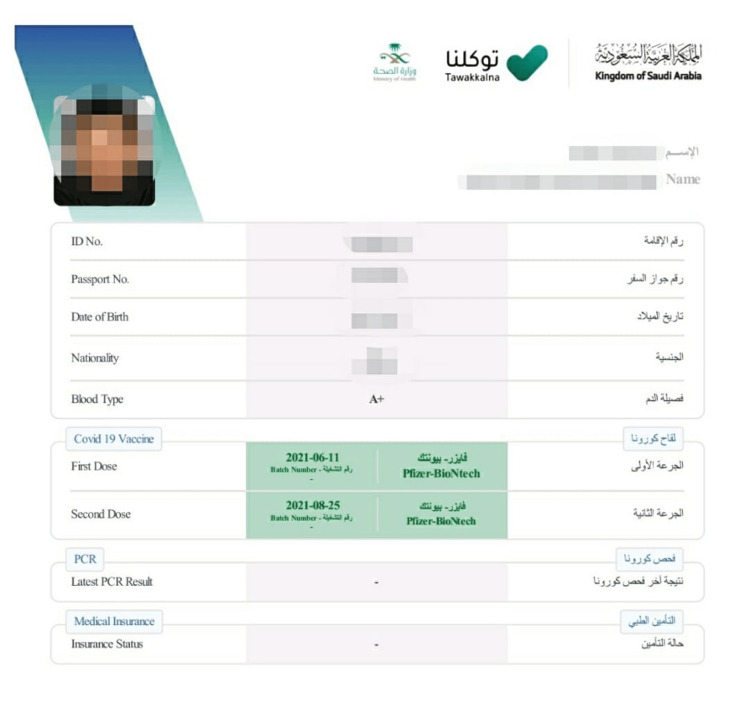
Sample electronic health passport

Soon after, the Tawakkalna application was developed by the Saudi Data and Artificial Intelligence Authority (SDAIA) in May 2020 which provides an array of electronic health services [7]. Services include COVID-19 test booking, COVID-19 vaccine booking, gathering permit during the lockdown, requests for Umrah and Hajj pilgrimage, health condition status, infection status (unidentified, infected, exposed, institutional quarantine, home quarantine, non-immune, incomplete vaccination, no record of infection, exempted and immune), ambulance request, and generating an electronic health passport (Figure 1). In addition, Tawakkalna provides other civil services such as digital government documents (Nationaldriver'sriver license, vehicle registration) and personal information (name, age, blood group, national address).

In June 2020, the Tabaud application was developed by SDAIA as a contact tracing service. It notifies users whenever they get within the proximity of any contacted or confirmed COVID-19 individuals. Tabaud relies on Global Positioning System (GPS) and Bluetooth technologies to detect any smartphones within range and ensure that the user is not exposed to any confirmed COVID-19 cases. Similar applications using similar technology such as TraceTogether in Singapore also used Bluetooth to trace the contact of confirmed COVID-19 cases [8]. Similar technologies were then adapted widely in other countries including Ehteraz in Qatar, Aarogya Setu in India, COVID Safe in Australia, and others [9-11].

The MOH’s previous experience with applications and services before the COVID-19 pandemic paved the way for the rapid implementation of the applications and services during the pandemic. For example, the Mawid application served as the backbone of the electronic healthcare system provided by the MOH before the pandemic and was used by patients for booking, canceling, and rescheduling appointments in primary healthcare centers [12]. The application also provides some extra services such as finding the nearest healthcare center and a self-assessment tool for COVID-19 symptoms. User satisfaction was reported by Alanzi et al. and showed that participants were highly satisfied with the ease of use and value of the application, especially during the pandemic [13]. Furthermore, the National Single Sign-On service has been implemented prior to the pandemic and helped in providing user access to all smartphone applications during the pandemic. 

Saudi Arabia’s experience with mass vaccination was enhanced with the rapid implementation of policies that ensure the availability of vaccine products to the community, raise vaccine awareness, and encourage the public to receive them. COVID-19 vaccine was managed using two main smartphone applications; Tawakkalna and Sehaty besides other complimentary services. The vaccine acceptance was studied by Al-Mohaithef et al., whoh demonstrated that 64.7% of participants are willing to take the vaccine due to a host of factors including highly placed trust in the healthcare system [14].

## Conclusions

The digitalization of healthcare services in Saudi Arabia has eased the communication between the public and healthcare professionals. Furthermore, electronic health services served as an effective tool against the spread of the pandemic and helped in reducing the number of COVID-19 cases and deaths by promoting and easing vaccination and testing processes. Ease of use and access has improved the adaptability of these tools among the public. As these tools become an integral element of healthcare, further evaluation of their acceptance among the public as well as areas of improvement of each tool is warranted.
